# Low-grade fibromyxoid and sclerosing epithelioid fibrosarcoma: distinct behaviour in the same spectrum of ultra-rare sarcomas

**DOI:** 10.3389/fonc.2026.1943496

**Published:** 2026-10-15

**Authors:** Beatriz Castanheira, Inês Soldin, Joana Vieira, Mariana Afonso, Sofia Conde, Augusto Moreira, Miguel Henriques Abreu

**Affiliations:** 1 Medical Oncology Department, Hospital Garcia de Orta, Almada, Portugal; 2 Medical Oncology Department, Instituto Português de Oncologia do Porto Francisco Gentil, Oporto, Portugal; 3 Genetics Department, Instituto Português de Oncologia do Porto Francisco Gentil, Oporto, Portugal; 4 Pathology Department, Instituto Português de Oncologia do Porto Francisco Gentil, Oporto, Portugal; 5 Radiation Oncology Department, Instituto Português de Oncologia do Porto Francisco Gentil, Oporto, Portugal; 6 Surgery Department, Instituto Português de Oncologia do Porto Francisco Gentil, Oporto, Portugal

**Keywords:** *EWSR1*, *FUS*, LGFMS, sarcoma, SEF, hybrid tumours, ultra-rare sarcoma

## Abstract

**Introduction:**

Low-grade fibromyxoid sarcoma (LGFMS) and sclerosing epithelioid fibrosarcoma (SEF) represent a molecularly related spectrum of ultra-rare soft-tissue sarcomas characterised by recurrent *FUS* or *EWSR1* rearrangements and frequent *MUC4* expression. Despite sharing recurrent molecular alterations, their clinical behaviour differs markedly, positioning them at opposite ends of a biologically related spectrum with distinct clinical trajectories. This study aimed to characterise the clinicopathological features, molecular profiles, treatment approaches, and outcomes across this spectrum.

**Methods:**

We retrospectively reviewed consecutive patients with LGFMS or SEF at a national EURACAN-designated sarcoma reference centre between 2013 and 2024. Clinicopathological characteristics, molecular findings, treatments, and outcomes were collected and analysed descriptively.

**Results:**

Twenty patients were included: 11 (55%) LGFMS, six (30%) SEF and three (15%) hybrid tumours. *MUC4* was positive in 13 cases (65%). Molecular analysis was informative in 19 patients (95%), with *FUS* or *EWSR1* rearrangements in 16 (84%). *FUS* predominated (63%); *EWSR1* rearrangements were confined to SEF (21%). Nineteen patients (95%) underwent curative-intent surgery. Local recurrence occurred in two (10%). Distant metastases developed in three (15%), all with SEF or hybrid histology. Metastatic progression was early (median 6 months), associated with high-risk histological features and *EWSR1* rearrangement. SEF accounted for the only disease-related death; median overall survival was not reached, with 95% of patients alive at last follow-up.

**Discussion:**

These findings highlight the divergent clinical behaviour across the LGFMS–SEF spectrum and support closer surveillance of high-risk tumours.

## 1 Introduction

Low-grade fibromyxoid sarcoma (LGFMS) and sclerosing epithelioid fibrosarcoma (SEF) are ultra-rare malignant soft-tissue tumours that encompass a biologically related pathological and molecular spectrum, although their precise nosological relationship — a single disease continuum versus two distinct but related entities — remains a matter of ongoing debate. Although LGFMS is traditionally described as a low-grade tumour with an understated microscopic appearance, long-term series have shown that it carries a risk of late local recurrence and distant metastasis, sometimes emerging decades after the initial diagnosis (1–3). SEF, initially considered a distinct entity, is now recognised as a biologically related entity within this spectrum, characterised by increased cellular density, more pronounced cytological atypia, and a clearly more aggressive clinical course (4, 5).

An intermediate or hybrid form, displaying overlapping histological features of both LGFMS and SEF, has also been increasingly recognised. These tumours often show areas typical of LGFMS admixed with more cellular, sclerosing or epithelioid regions reminiscent of SEF, and frequently harbour *FUS::CREB3L2, FUS::CREB3L1*, or occasionally *EWSR1-*related fusions (6–8). Emerging evidence suggests that these hybrid LGFMS/SEF tumours may exhibit a clinical behaviour closer to SEF, including a higher propensity for recurrence and metastatic progression, thereby positioning them within the higher-risk segment of this biological spectrum (7, 8).

Despite their rarity, these tumours pose significant clinical challenges. LGFMS may initially present with slow growth and minimal symptoms, thereby delaying diagnosis (1, 9). SEF, conversely, is associated with earlier recurrence, faster metastatic progression and poorer prognosis, particularly when adverse pathological or molecular features are present (4, 5, 10). Given the paucity of robust evidence and the prolonged natural history of these neoplasms, institutional series from specialised sarcoma centres remain essential for improving understanding of clinical behaviour, refining prognostic assessment and informing patient management. Therefore, this study aims to characterise the clinicopathological features, molecular profiles, treatment approaches, and long-term outcomes in a cohort of patients diagnosed with LGFMS, SEF, and related tumours at a comprehensive cancer centre specialising in sarcomas.

## 2 Materials and methods

Retrospective study of all patients diagnosed with LGFMS or SEF between August 2013-May 2024 [median follow-up 66 months (20–164)] at Instituto Português de Oncologia do Porto, Francisco Gentil, EPE, the Portuguese major Comprehensive Cancer Centre (national reference centre and EURACAN member for soft tissue sarcomas). All diagnoses were validated by a pathologist with expertise in sarcomas. Clinical, pathological and molecular information was retrieved from electronic medical records, including demographics, diagnostic approach, tumour characteristics, treatment details and follow-up. Histological assessment focused on key features such as cellularity, stromal pattern, atypia, mitotic activity and surgical margins. MUC4 immunohistochemistry was recorded as positive or negative. Molecular studies were performed using fluorescence *in situ* hybridisation (FISH) with break-apart probes for *FUS* and/or *EWSR1*, and/or a targeted next-generation sequencing (NGS) sarcoma fusion panel that routinely screens for gene rearrangements involving *FUS, EWSR1, CREB3L1*, and *CREB3L2*.

The primary outcomes were local recurrence and overall survival, defined as the time from initial diagnosis to death from any cause; secondary outcomes included time to metastatic progression and associations with prognostic values of pathology or molecular features. Descriptive statistics were used to summarise all variables, with categorical data presented as frequencies and percentages and continuous data expressed as median and interquartile range. No inferential statistical tests were performed, given the small sample size and the limited number of outcome events. Statistical analysis was conducted using IBM SPSS Statistics (version 27). This observational study is reported in accordance with the STROBE (Strengthening the Reporting of Observational Studies in Epidemiology) guidelines for cohort studies (**Supplementary Table S1**). The study was conducted in accordance with the Declaration of Helsinki; as a retrospective analysis of routinely collected, anonymised clinical data, it did not require formal ethics committee approval or additional informed consent (see Ethics statement).

## 3 Results

During the analysed period, 20 consecutive patients were treated at our institution and were all included.

### 3.1 Patient and tumour characteristics

Demographic, pathologic and molecular characteristics of the cohort were summarised in **Table 1**.

**Table 1:** Sample characterisation.

Variable	LGFMS (n=11, %)	SEF (n=6, %)	Hybrid (n=3, %)	Overall (n=20, %)
Gender Male Female	6 (54.5) 5 (45.5)	2 (33.3) 4 (66.7)	2 (66.7) 1 (33.3)	10 (50.0) 10 (50.0)
Age at diagnosis (y), median (IQR)	39 (31)	41 (24)	43 (36)	41 (29)
Histopathologic features				
Hypercellularity Atypia >4 mitoses/10 high-power fields	8 (72.7) 1 (9.1) 4 (36.4)	6 (100.0) 5 (83.3) 5 (83.3)	3 (100.0) 2 (66.7) 3 (100.0)	17 (85.0) 8 (40.0) 12 (60.0)
MUC4 expression (%) Positive	5 (45.5)	6 (100.0)	2 (66.7)	13 (65.0)
Molecular features				
Number of patients with informative molecular analyses (%)^a^	11 (100.0)	5 (83.3)	3 (100.0)	19 (95.0)
*FUS* + *FUS* rearrangement (FISH) *FUS::CREB3L2* (NGS)	**10 (90.9)** 9 (90.0) **2 (100.0)**	0 (0) 0 (0) 0 (0)	2 (66.7) **2 (100.0)** 1 (50.0)	12 (63.2) **11 (73.3)** **3 (33.3)**
*EWSR1* + *EWSR1* rearrangement (FISH) *EWSR1::CREB3L1* (NGS) *EWSR1::CREB3L2* (NGS)	0 (0) 0 (0) 0 (0) 0 (0)	**4 (80.0)** **4 (80.0)** **1 (20.0)** **2 (40.0)**	0 (0) 0 (0) 0 (0) 0 (0)	4 (21.1) **4 (66.7)** **1 (11.1)** **2 (22.2)**
Primary site (%) Extremities Trunk Other	6 (54.5) 4 (36.4) 1 (9.1)	2 (33.3) 3 (50.0) 1 (16.7)	2 (66.7) 1 (33.3) 0 (0)	10 (50.0) 8 (40.0) 2 (10.0)
Treatments of primary disease Surgery (N = 19, %) Yes R0 R1	11 (100.0) 10 (90.9) 1 (9.1)	5 (83.3) 2 (40.0) 3 (60.0)	3 (100.0) 3 (100.0) 0 (0)	19 (95.0) 15 (78.9) 4 (21.1)
Radiotherapy (N = 5, %) Radical Adjuvant Palliative	0 (0) 1 (9.1) 0 (0)	1 (16.7) 2 (33.3) 1 (16.7)	0 (0) 0 (0) 0 (0)	1 (5.0) 3 (15.0) 1 (5.0)
Systemic therapy (N = 1, %) Palliative	0 (0)	1 (16.7)	0 (0)	1 (5.0)

^a^For “^a^Number of patients with informative molecular analyses”, the denominator is the total number of patients per histological subtype (N = 11, 6 and 3 for LGFMS, SEF and hybrid tumours, respectively). For “FUS+” and “EWSR1+”, the denominator is the number of patients with informative molecular analyses within each subtype (11, 5 and 3 for LGFMS, SEF and hybrid tumours, respectively, reflecting the single non-informative SEF case). For the method-specific rows — “FUS rearrangement (FISH)”, “FUS::CREB3L2 (NGS)”, “EWSR1 rearrangement (FISH)”, “EWSR1::CREB3L1 (NGS)” and “EWSR1::CREB3L2 (NGS)” — the denominator is the number of patients tested by that specific method within each subtype, not the subtype total.

Values in bold indicate the molecular findings in which a FUS or EWSR1 rearrangement or fusion was detected; values not in bold represent absence of detection (n = 0).

In our cohort, there was no gender predominance. LGFMS were more frequent than SEF (11 [55%] vs 6 [30%] cases). There were three cases of hybrid histology (15%).

Molecular analysis was performed in all patients, of whom 19 (95%) had an informative result; rearrangement of *FUS* or *EWSR1* was found in 16 (84%). *FUS* rearrangements predominated overall (12, 63%), and *EWSR1* rearrangements were confined to SEF (4, 21%) (**Table 1**, **Figure 1**). It is noteworthy that among patients harbouring *FUS* rearrangements analysed by NGS, the *FUS::CREB3L2* fusion was identified. Regarding *EWSR1* rearrangements detected in cases of SEF and hybrid histology, two patients harboured the *EWSR1::CREB3L2* fusion, while one presented the *EWSR1::CREB3L1* fusion.

**Figure 1:**
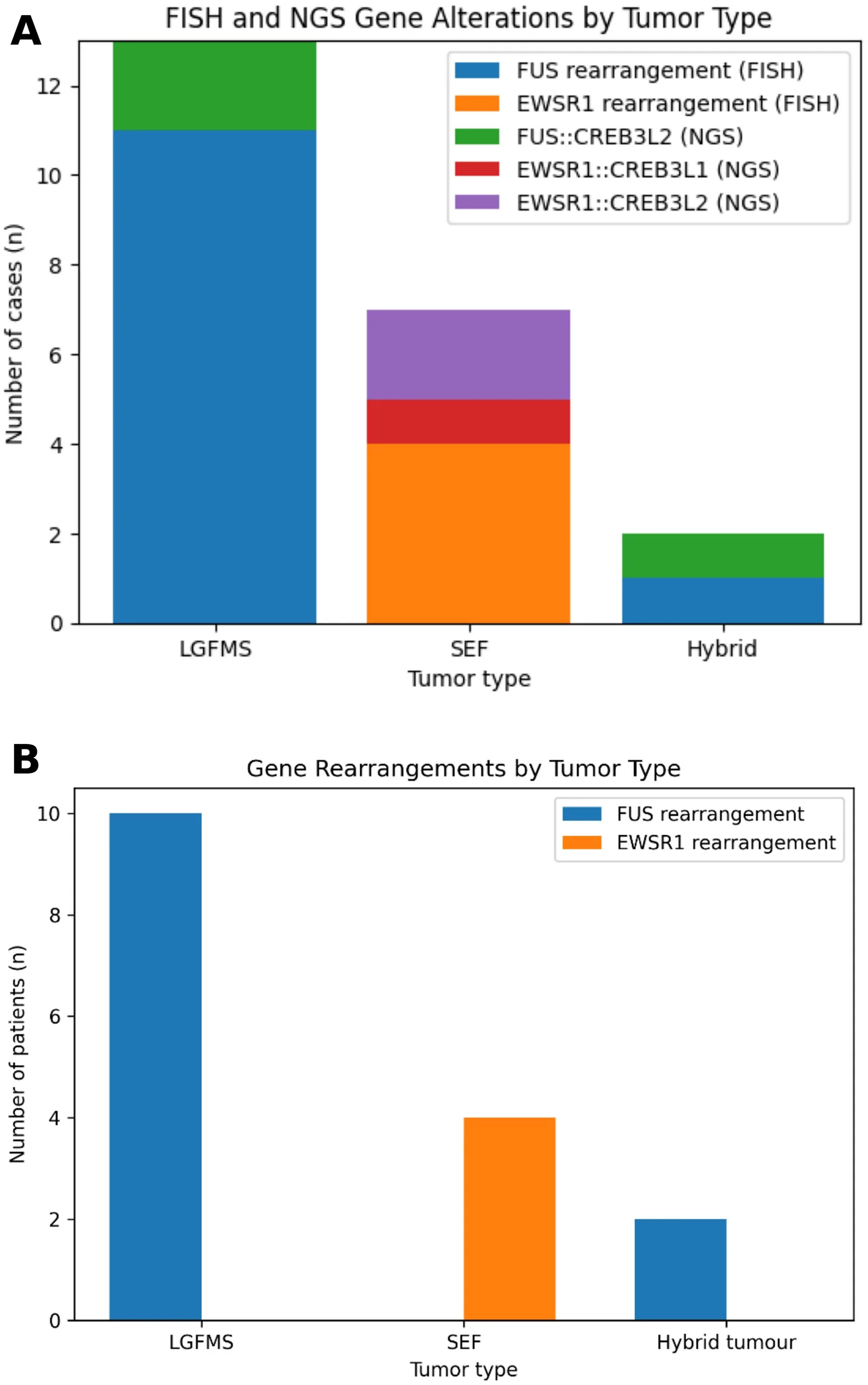
Molecular rearrangements by tumour type (n = 19 patients with informative molecular analyses). **(A)** FISH and NGS gene alterations by tumour type. *FUS* and *EWSR1* rearrangements were assessed by FISH, while specific gene fusions were identified by NGS. Stacked bar chart showing the absolute number of patients by tumour type (LGFMS, SEF, and hybrid tumour) and specific molecular alteration detected by fluorescence *in situ* hybridisation (FISH) or next-generation sequencing (NGS). *FUS* rearrangements detected by FISH predominated in LGFMS (n = 10 of 10 informative cases). In SEF, *EWSR1* rearrangements were identified in 4 of 6 patients, including *EWSR1::CREB3L2* (n = 2) and *EWSR1::CREB3L1* (n = 1) fusions by NGS, and one case detected by FISH only. In hybrid tumours, *FUS* rearrangements were present in 2 of 3 cases, one identified by FISH and one as *FUS::CREB3L2* by NGS. **(B)** Overall gene rearrangements by tumour type. Bar chart summarising the total frequency of *FUS* (n = 12; 63%) and *EWSR1* (n = 4; 21%) rearrangements across histological subtypes. *FUS* rearrangements were identified in LGFMS (n = 10) and hybrid tumours (n = 2), whereas *EWSR1* rearrangements were confined exclusively to SEF (n = 4). FISH, fluorescence *in situ* hybridisation; NGS, next-generation sequencing; LGFMS, low-grade fibromyxoid sarcoma; SEF, sclerosing epithelioid fibrosarcoma.

Regarding the extent of molecular testing, FISH alone was performed in 11 patients (55.0%), NGS alone in 2 (10.0%), and both techniques in 7 (35.0%); overall, FISH was performed in 18 patients (90.0%) and NGS in 9 patients (45.0%). By histological subtype, in LGFMS (n=11) FISH alone was performed in 9 patients (81.8%), NGS alone in 1 (9.1%), and both in 1 (9.1%); in SEF (n=6), FISH alone was performed in 1 patient (16.7%), NGS alone in 1 (16.7%), and both in 4 (66.7%); and in hybrid tumours (n=3), FISH alone was performed in 1 patient (33.3%) and both techniques in 2 (66.7%), with no patient tested by NGS alone. One SEF patient underwent both FISH and NGS but had non-diagnostic results with both techniques, accounting for the single non-informative case in the overall cohort (1/20, 5.0%).

### 3.2 Treatment

Nineteen patients (95%) underwent surgical resection with curative intent, reflecting the predominantly localized and resectable presentation of the cohort. Surgical treatment was selected according to tumour location and resectability, with the aim of achieving complete tumour excision and negative margins whenever feasible. Radical-intent radiotherapy was administered to one patient (5%) and adjuvant radiotherapy to three patients (15%), based on individual multidisciplinary assessment and the presence of high-risk pathological features and/or close or compromised margins (**Table 1**). Treatment was delivered using either volumetric modulated arc therapy (VMAT) or three-dimensional conformal radiotherapy (3D-CRT) with multileaf collimator (MLC) beam shaping, to a total dose of 66–70 Gy in 33–35 fractions over 6.5–7 weeks. The patient with SEF histology who presented with bone metastasis at diagnosis underwent bone-directed radiotherapy with palliative intent. Besides radiotherapy, this patient received first-line systemic palliative chemotherapy consisting of six cycles of doxorubicin at a dose of 75 mg/m², discontinued due to progressive bone disease. It was followed by three cycles of etoposide (100 mg/m^2^) in association with iphosphamide (1800 mg/m²), permanently suspended due to new lung and liver lesions and progressive bone disease. Pazopanib (800 mg daily) was used as third-line, discontinued after three months due to progressive disease.

### 3.3 Follow-up and survival outcomes

After a median follow-up of 65 months, local recurrence occurred in two patients (10%), with a median time to recurrence of 32 months (**Table 2**). One case occurred in a patient with SEF and the other in a patient with a hybrid tumour. They were both associated with adverse pathological features, such as cellularity, atypia, and a higher mitotic count.

**Table 2:** Clinical outcomes according to histology.

Outcome	LGFMS (n=11)	SEF (n=6)	Hybrid (n=3)	Overall (n=20)
Median follow-up (months)	71	47	53	65
Local recurrence (n, %)	0	1 (16.7)	1 (33.3)	2 (10.0)
Median time to LR (months)	NA	48	16	32
Distant metastasis (n, %)	0	2 (33.3)	1 (33.3)	3 (15.0)
Median time to DM (months)	NA	6	0	6
Status at last follow-up (%) Alive, No evidence of disease Alive, With evidence of disease Dead	11 (100.0) 0 (0) 0 (0)	5 (83.3) 0 (0) 1 (16.7)	3 (100.0) 0 (0) 0 (0)	19 (95.0) 0 (0) 1 (5.0)

The table summarises median follow-up duration, incidence of local recurrence (LR) and distant metastasis (DM), median time to recurrence or metastasis, and status at last follow-up. Data are presented as median (months) or number (%), as appropriate. NA, not applicable.

Distant metastases developed in three patients (15%), two with SEF —one with bone metastases and the other with bone, lung and liver disease – and one with hybrid histology, who progressed with lung metastasis. The observed median time to distant metastasis was 6 months (**Table 2**). All cases were associated with the previously referred high-risk pathological features, and the two SEF also presented *EWSR1* rearrangement.

At the last follow-up, all patients with LGFMS were alive and had no evidence of disease. The patient with hybrid histology who developed lung metastases underwent complete metastasectomy and remained disease-free thereafter (46 months). One patient with SEF died due to disease progression (5%). Overall survival was 95% (19/20): LGFMS and hybrid 100% survival (11/11; 3/3), SEF 83% survival (5/6). Owing to the limited number of events, median overall survival was not reached during the study period (August 2013 - January 2026) (**Table 2**).

## 4 Discussion

Consistent with the findings of the recent international multicentre study published in the *American Journal of Surgical Pathology* (11), our single-centre experience confirms that LGFMS have a more indolent clinical behaviour than SEF. In our cohort, metastatic progression occurred in patients with SEF and, in one case, hybrid histology, and was associated with adverse pathological and molecular features, reinforcing previously reported differences in survival and disease trajectory between histological subtypes. These findings support the concept that, although LGFMS and SEF may lie along a biologically related spectrum, they differ substantially in clinical course. Notably, although hybrid tumours share a greater molecular overlap with LGFMS, the clinical behaviour observed in our series — including both local recurrence and distant metastasis — together with data from the cited literature, places them closer to the higher-risk group represented by SEF. We therefore suggest that hybrid tumours be stratified clinically as high-risk, in line with SEF, irrespective of their predominant molecular profile. The concentration of relapse events within the SEF and hybrid subgroups in our series mirrors observations from larger international datasets (16, 17), further emphasising the prognostic implications of histological classification within this tumour spectrum.

The molecular findings in our cohort argue against a true biological continuum between LGFMS and SEF, instead supporting relatively distinct molecular profiles within a related tumour spectrum, with potential prognostic implications. *FUS* rearrangements predominated among LGFMS, whereas SEF showed *EWSR1* rearrangements, the latter often associated with more aggressive disease (7, 9, 12). However, this association should be regarded as an observation requiring validation in larger, multicentre cohorts rather than as an established prognostic factor. The presence of an *EWSR1* rearrangement in one patient who developed metastases aligns with previous reports suggesting that *EWSR1*-positive tumours may represent a particularly high-risk subgroup (12, 13). However, in our cohort this association represents a hypothesis-generating observation, and its prognostic significance requires confirmation in larger multicentre series. MUC4 expression, identified in most cases, remains a highly reliable diagnostic marker across tumour types, consistent with its recognised sensitivity and specificity (6). The lower overall MUC4 positivity observed in our cohort (65%), driven mainly by the LGFMS subgroup, likely reflects methodological factors related to retrospective, binary scoring over a prolonged inclusion period and the focal nature of MUC4 expression in some LGFMS, rather than a true reduction in diagnostic sensitivity. Taken together, the near-complete molecular exclusivity observed between LGFMS and SEF — with *FUS* rearrangements confined to LGFMS/hybrid tumours and *EWSR1* rearrangements confined to SEF — indicates that, despite occasional histological overlap (particularly in hybrid tumours), these entities are molecularly distinct rather than representing a single biological continuum.

The role of systemic therapy remains limited. Only one patient in our cohort received chemotherapy, reflecting current evidence and understanding that conventional sarcoma regimens show modest benefit in LGFMS and SEF (14). Radiotherapy was used selectively, primarily in the presence of high-risk pathological features or close margins, which is consistent with international guidelines (14). The predominance of R0 resections in our series likely contributed to the overall favourable outcomes in the LGFMS group, supporting the importance of complete surgical excision as the main treatment.

The recurrence and metastasis rates observed in our study are broadly comparable to those described in the literature, although the relatively small sample size limits the precision of these estimates. Long-term follow-up is essential, as several studies have documented recurrences and metastases many years—and occasionally decades— after the initial diagnosis (2, 3, 15). Our median follow-up of 65 months, more than 5 years, provides some insight into mid-term outcomes but may not fully capture the late events that characterise these tumours. The particularly short interval to metastatic progression in the SEF subgroup (median six months) echoes earlier observations of this tumour’s more aggressive nature and highlights the importance of early multidisciplinary discussion and appropriate imaging surveillance.

Like most studies of ultra-rare sarcomas, our work is limited by a small sample size. While the results align closely with international evidence, the limited number of events precludes a formal statistical comparison across subgroups. Nevertheless, small, high-quality series from expert centres with follow-up longer than 5 years remain crucial for refining clinical understanding, especially in European populations, where published experience is scarce. Collaborative multicentre efforts — particularly those involving consistent molecular assessment — will be essential to improve prognostic stratification and guide future therapeutic developments.

Relative to the existing literature, this study contributes in several respects: (i) it is one of the few European series with systematic and concordant FISH and NGS testing for *FUS*/*EWSR1* rearrangements; (ii) it provides a median follow-up exceeding five years (65 months), which is particularly relevant given the late-onset behaviour of these tumours; and (iii) it derives from a EURACAN reference centre with dedicated multidisciplinary diagnostic validation, a setting under-represented in the current literature. **Table 3** summarises the histological distribution, molecular findings, recurrence/metastasis rates, and survival outcomes of our series alongside previously published cohorts, to contextualise these contributions relative to the existing literature.

**Table 3:** Comparative summary of previously published series alongside the present study.

Study	n (LGFMS/SEF/Hybrid)	Design	Molecular findings	Recurrence/Metastasis	Survival outcomes
Blay et al. (17), Eur J Cancer 2024	330 (257/73/—)	French nationwide registry (NETSARC+), retrospective	Not reported	Metastasis at diagnosis: SEF 30% vs LGFMS 4% (p<0.0001); more visceral, bone and trunk primary sites in SEF	Not reported in available abstract
Giani et al. (11), Am J Surg Pathol 2025	294 (239/32/23)	International multicentre, 27 institutions/14 countries, primary localised disease treated with curative intent	Not the primary focus of this study	Secondary endpoints: cumulative incidence of local recurrence and distant metastases (not further specified in available abstract)	Median follow-up 57.1 months; 12/294 (4.1%) deaths overall
Giani et al. (16), ESMO Open 2024	Not specified in available abstract	Retrospective, 28 global centres, focused on advanced/metastatic disease	Not the primary focus of this study	Focus on outcomes and systemic treatment specifically in advanced/metastatic LGFMS, SEF and hybrid tumours	Post-metastasis survival reported (specific figures not available from the published abstract)
Present study, 2026	20 (11/6/3)	Single EURACAN reference centre, retrospective, concordant FISH and NGS testing	FUS confined to LGFMS/hybrid; EWSR1 confined to SEF (near-complete molecular exclusivity)	Local recurrence 10% (2/20); distant metastasis 15% (3/20), all in SEF or hybrid tumours	Median follow-up 65 months; overall survival 95% (19/20); 1 SEF disease-related death

Methodological limitations should also be acknowledged. The retrospective, single-centre design and the small sample size may introduce selection and referral bias and limit the generalisability of our findings. The limited number of metastatic events and disease-related deaths substantially restricts statistical power and precludes robust prognostic comparisons between histological and molecular subgroups. Finally, the absence of external validation limits the ability to generalise our observations to other populations. These findings should therefore be interpreted as hypothesis-generating and require validation in larger, multicentre cohorts with longer follow-up and standardised molecular and clinical assessment.

In summary, our findings add further support to the notion that LGFMS and SEF, although related, may differ substantially in clinical behaviour. In our cohort, SEF was associated with metastatic progression and adverse pathological and molecular features, consistent with findings from larger international series. However, given the retrospective, single-centre design, the limited number of events, and the small number of hybrid LGFMS/SEF cases, these observations should be interpreted cautiously, considered hypothesis-generating, and cannot, on their own, support definitive management recommendations; treatment decisions should continue to be individualised through multidisciplinary sarcoma tumour board discussion. SEF, especially when displaying adverse pathological (>4 mitoses/10 high-power fields, hypercellularity, nuclear atypia, tumour necrosis and dedifferentiation) or molecular features (specific gene fusions involving *FUS* or *EWSR1* genes), may represent a higher-risk subgroup warranting close and sustained follow-up. Continued international collaboration will be vital to further clarify the prognostic implications of histological and molecular features across this tumour spectrum.

## Data Availability

The datasets presented in this article are not readily available because patient-level clinicopathological and molecular data are subject to ethical, privacy and institutional data protection constraints, and sharing them would risk re-identification of individuals in this small cohort of ultra-rare sarcoma patients. Requests to access the datasets should be directed to Beatriz Castanheira, biacastanheira11@gmail.com.
