# Supplementary material for: Low-grade fibromyxoid and sclerosing epithelioid fibrosarcoma: distinct behaviour in the same spectrum of ultra-rare sarcomas
Source: Front Oncol. 2026 Oct 15;16:1943496. doi: 10.3389/fonc.2026.1943496 (PMC13716661; doi:10.3389/fonc.2026.1943496)
Supplement: Supplementary file 1 [file Table1.docx]

**Supplementary Table S1. STROBE checklist for cohort studies**

*Low-grade fibromyxoid and sclerosing epithelioid fibrosarcoma: distinct behaviour in the same spectrum of ultra-rare sarcomas. Section references refer to the published article. Checklist adapted from von Elm E, et al. The Strengthening the Reporting of Observational Studies in Epidemiology (STROBE) statement. (2007).*

| **Item** | **Topic** | **STROBE recommendation (cohort studies)** | **Where reported / comment** |
| --- | --- | --- | --- |
| **1a** | Title and abstract | Indicate the study’s design with a commonly used term in the title or the abstract | Abstract (Methods): consecutive patients “retrospectively reviewed”; Methods, paragraph 1: “Retrospective study” |
| **1b** |  | Provide in the abstract an informative and balanced summary of what was done and what was found | Abstract (structured: Introduction, Methods, Results, Discussion) |
| **2** | Background/rationale | Explain the scientific background and rationale for the investigation being reported | Introduction, paragraphs 1–3 |
| **3** | Objectives | State specific objectives, including any prespecified hypotheses | Introduction, last paragraph; Abstract. Descriptive study; no prespecified hypotheses |
| **4** | Study design | Present key elements of study design early in the paper | Methods, paragraph 1 |
| **5** | Setting | Describe the setting, locations, and relevant dates, including periods of recruitment, exposure, follow-up, and data collection | Methods, paragraph 1 (Instituto Português de Oncologia do Porto; diagnoses August 2013–May 2024); Results 3.3 (follow-up to January 2026) |
| **6a** | Participants | Give the eligibility criteria, and the sources and methods of selection of participants. Describe methods of follow-up | Methods, paragraph 1 (all patients diagnosed with LGFMS or SEF; diagnoses validated by an expert sarcoma pathologist; electronic medical records); Results 3.3 |
| **6b** |  | For matched studies, give matching criteria and number of exposed and unexposed | Not applicable (no matching) |
| **7** | Variables | Clearly define all outcomes, exposures, predictors, potential confounders, and effect modifiers. Give diagnostic criteria, if applicable | Methods, paragraphs 1–2 (demographics, tumour characteristics, MUC4 immunohistochemistry, FISH/NGS, treatment, primary and secondary outcomes) |
| **8** | Data sources/measurement | For each variable of interest, give sources of data and details of methods of assessment (measurement) | Methods, paragraph 1 |
| **9** | Bias | Describe any efforts to address potential sources of bias | Methods, paragraph 1 (consecutive inclusion of all patients; expert pathological validation); Discussion (selection and referral bias; retrospective binary MUC4 scoring) |
| **10** | Study size | Explain how the study size was arrived at | Results (20 consecutive patients, all included); Discussion. Size determined by the number of eligible patients (ultra-rare disease); descriptive study |
| **11** | Quantitative variables | Explain how quantitative variables were handled in the analyses. If applicable, describe which groupings were chosen and why | Methods, paragraph 2 (continuous data as median and interquartile range); Tables 1 and 2 |
| **12a** | Statistical methods | Describe all statistical methods, including those used to control for confounding | Methods, paragraph 2 (descriptive statistics; IBM SPSS Statistics version 27). No confounding control (no inferential analysis) |
| **12b** |  | Describe any methods used to examine subgroups and interactions | Results and Tables 1–2 (descriptive, by histological subtype). No interaction analysis |
| **12c** |  | Explain how missing data were addressed | Results 3.1 (molecular analysis informative in 19/20; one non-informative case reported) |
| **12d** |  | If applicable, explain how loss to follow-up was addressed | Status at last follow-up reported for all 20 patients (Results 3.3, Table 2) |
| **12e** |  | Describe any sensitivity analyses | Not applicable |
| **13a** | Participants | Report numbers of individuals at each stage of the study | Results (20 consecutive patients treated and all included) |
| **13b** |  | Give reasons for non-participation at each stage | Not applicable (all eligible patients included) |
| **13c** |  | Consider use of a flow diagram | Not used (single-stage inclusion; all 20 patients included) |
| **14a** | Descriptive data | Give characteristics of study participants and information on exposures and potential confounders | Table 1; Results 3.1–3.2 |
| **14b** |  | Indicate number of participants with missing data for each variable of interest | Results 3.1 (one non-informative molecular result) |
| **14c** |  | Summarise follow-up time (e.g., average and total amount) | Results 3.3 (median follow-up 65 months); Table 2 |
| **15** | Outcome data | Report numbers of outcome events or summary measures over time | Results 3.3; Table 2 |
| **16a** | Main results | Give unadjusted estimates and, if applicable, confounder-adjusted estimates and their precision | Not applicable: descriptive analysis only (Methods, paragraph 2) |
| **16b** |  | Report category boundaries when continuous variables were categorised | Not applicable |
| **16c** |  | If relevant, consider translating estimates of relative risk into absolute risk for a meaningful time period | Not applicable |
| **17** | Other analyses | Report other analyses done (e.g., subgroup analyses, sensitivity analyses) | Subgroup description by histological subtype only (Tables 1–2) |
| **18** | Key results | Summarise key results with reference to study objectives | Abstract; Discussion, paragraph 1 |
| **19** | Limitations | Discuss limitations of the study, taking into account sources of potential bias or imprecision. Discuss both direction and magnitude of any potential bias | Discussion (methodological limitations) |
| **20** | Interpretation | Give a cautious overall interpretation of results considering objectives, limitations, multiplicity of analyses, results from similar studies, and other relevant evidence | Discussion, final paragraphs; Table 3 |
| **21** | Generalisability | Discuss the generalisability (external validity) of the study results | Discussion (single-centre design; need for external validation) |
| **22** | Funding | Give the source of funding and the role of the funders for the present study | Funding section (no external funding) |
